# Diagnosis Through Whole Genome Sequencing and Care Utilization in Children With Severe Illness

**DOI:** 10.1001/jamanetworkopen.2026.34372

**Published:** 2026-09-17

**Authors:** Joao M. L. Dias, Ravi P. More, Duncan Butler, Julian Brown, Courtney E. French, Helen Dolling, F. Lucy Raymond, David H. Rowitch, Catherine E. Aiken

**Affiliations:** 1 Department of Paediatrics, University of Cambridge, School of Clinical Medicine, Cambridge, United Kingdom; 2 Prescribing Services Ltd, Norfolk, United Kingdom; 3 Litcham Health Centre, Norfolk, United Kingdom; 4 Children’s Rare Disease Collaborative, Boston Children’s Hospital, Boston, Massachusetts; 5 Department of Psychology, University of Cambridge, Cambridge, United Kingdom; 6 Department of Medical Genetics, University of Cambridge, School of Clinical Medicine, Cambridge, United Kingdom; 7 Departments of Pediatrics, Cedars-Sinai Guerin Children’s, Los Angeles, California; 8 Department of Obstetrics and Gynaecology, University of Cambridge, School of Clinical Medicine, Cambridge, United Kingdom

## Abstract

**Importance:**

Whole genome sequencing (WGS) is increasingly used to diagnose children with severe illness, yet the long-term association of a genetic diagnosis with health care utilization and resource allocation remains poorly understood.

**Objective:**

To assess the association of a genetic diagnosis through WGS with long-term health care utilization metrics in children and young people (hereafter *children*) with severe illness.

**Design, Setting, and Participants:**

This multicenter retrospective cohort study of children aged 0 to 18 years who were severely ill who underwent WGS used data from The Next Generation of Children Project (from December 2016 to August 2020) with medical record linkage and analysis of primary care medical records conducted between January 2022 and June 2024. The primary care and hospital medical records were linked using the UK National Institute for Health and Care Research Rare Disease BioResource, Cambridge.

**Exposures:**

Receipt of a genetic diagnosis compared with those who remained undiagnosed following WGS.

**Main Outcomes and Measures:**

The main outcome was a comparison of 36 health care utilization parameters, including hospitalizations, primary care prescriptions, and diagnostic tests.

**Results:**

Among the 270 children analyzed (mean [SD] age 8.65 [6.58-9.46] years; 149 males [55.2%]), those receiving a genetic diagnosis (87 [32.2%]) exhibited significantly higher overall health care utilization compared with undiagnosed peers (183 [67.8%]). This included an increase in median (IQR) hospital admissions (37 [18-66] vs 22 [10-35]) and more primary and secondary care outpatient visits each year (14 [7-26] vs 8 [4-13]), particularly for neurodevelopmental (annual treatment costs: £1280 [£507-£2529] vs £130 [£21-£354]) and seizure-related (annual treatment costs: £1277 [£320-£2271] vs £60 [£15-£143]) conditions. Children with genetic diagnoses received a median (IQR) higher volume of neurological (48 [22-88] vs 0) and gastrointestinal (5 [1-18] vs 0 [0-2])prescriptions. Median (IQR) differences specifically in neurodevelopmental (neurological prescriptions: 42 [22-64] vs 0 [0-3]) and pediatric intensive care unit (total cost prescriptions: £1045 [£244-£1914] vs £38 [£14-£220]) settings were observed. While a genetic diagnosis was associated with sustained and intensive health care utilization during the study period, it was also associated with a shift toward targeted, condition-specific medical care.

**Conclusions and Relevance:**

In this cohort study, a WGS diagnosis was associated with the integration of specialist care and the alignment of health care resources to support specific needs of children with complex disorders. These findings suggest that while longitudinal health care utilization remains intensive following a genetic diagnosis, identifying these conditions is important for accurately mapping and managing the downstream clinical resource requirements of this population.

## Key Points

**Question:**

Is a genetic diagnosis through whole genome sequencing associated with long-term health care utilization and cost savings in children and young people with severe illness?

**Findings:**

In this cohort study of comprehensively linked community medical records of 270 children and young people who received whole genome sequencing for rare genetic conditions, significantly higher health care utilization, including more hospitalizations and targeted prescriptions, were found in those who received a diagnosis.

**Meaning:**

The findings of this study suggest that a genetic diagnosis through whole genome sequencing is associated with sustained, high-intensity long-term health care utilization, which may facilitate a more precise and tailored alignment of clinical resources to the patient’s individual needs.

## Teaser

This cohort study assesses the association of a genetic diagnosis through whole genome sequencing with long-term health care utilization metrics among children and young people with severe illness.

## Introduction

Children admitted to a neonatal intensive care unit (NICU) or a pediatric intensive care unit (PICU) often present with severe, life-threatening conditions affecting survival and long-term neurodevelopment.^1,2,3^ Genetic disorders are recognized as leading contributors to morbidity and mortality, affecting 10% to 30% of children admitted to a NICU or a PICU.^4,5,6,7,8,9,10,11^ Whole genome sequencing (WGS) is thus increasingly common in clinical practice, particularly for suspected rare or monogenic diseases.^6,12,13,14,15,16^

Prior health economic analyses show that WGS reduces costs in the PICU, largely due to reducing acute hospital stays and avoiding invasive procedures.^2,3,4,8,17,18,19,20^ Initiatives such as Project Baby Bear in California report short-term net health care savings of more than $14 000 per infant.^21^ Unlike other trials (eg, GEMINI,^22^ NICUSeq^23^) or counterfactual studies (eg, Project Baby Bear,^21^ Project Baby Deer^24^) that evaluate WGS as a clinical intervention, our observational analysis compares long-term health care utilization in children and young people (hereafter *children*) with an identifiable genetic diagnosis vs those without the diagnosis. We mapped baseline differences in clinical natural history and resource demands between children with monogenic diagnoses vs those with multifactorial or environmental causes for admission. In contrast to studies of short-term financial benefits, the long-term implications of a genomic diagnosis for health care utilization remain underexplored, particularly in the UK National Health Service (NHS).^25,26^ We address this knowledge gap by linking pediatric WGS results to clinical outcome data provided by NHS Prescribing Services Ltd (Equality of Care Led Insights for Patient Safety & Engagement [ECLIPSE] Live), which comprises linked primary and secondary care data for more than 25 million patients.^27^

The Next Generation of Children Project (NGC), conducted in Cambridge (from December 2016 to August 2020), was an early demonstration of utility of WGS in the intensive care setting, with more than 90% of clinicians reporting improved confidence in patient management and family communication following testing.^28^ Initial evaluations within the NGC demonstrated an overall molecular diagnostic yield ranging from 21% to 45% via WGS.^12,28^ The NHS has subsequently provided rapid genome sequencing (R14 service) for children with acute illness who meet testing criteria,^29,30^ which has been shown to impact patient management and/or family reproductive counseling in nearly all diagnosed individuals.^25,31^

We incorporated detailed clinical phenotypes and NHS primary care medical records alongside genomic findings from the NGC. While we report accumulated costs, we conceptualized these as a standardized metric to quantify differences in long-term utilization rather than a formal health-economic or cost-effectiveness evaluation. Rather than assessing whether a genetic diagnosis was associated with health care utilization, our objective was to map and compare longitudinal clinical utilization patterns between children who were diagnosed and undiagnosed. We aimed to delineate baseline differences in clinical natural history and resource demands between children with and without identifiable genetic etiologies. Second, we evaluated the possibility that health care utilization and other clinical characteristics might be an independent means to identify children with a higher probability of a positive genetic result from WGS.

## Methods

### Research Governance and Ethics

The NGC study was approved by the Cambridge South Research Ethics Committee. Written informed consent was obtained from parents for diagnostic WGS and subsequent data linkage to medical records via the UK National Institute for Health and Care Research Rare Disease BioResource. The specific linkage to primary care data was approved by the NIHR BioResource Data Access Committee. This study followed the Strengthening the Reporting of Observational Studies in Epidemiology (STROBE) reporting guideline for cohort studies.

### Study Design and Participant Selection

The NGC study initially enrolled children from December 2016 to August 2020 across NICU and PICU, pediatric neurology, and genetics clinics.^12,28^ Enrollment required a high likelihood of an underlying monogenic condition based on clinical assessment.^32^ Included children presented with congenital anomalies, neurological symptoms, suspected metabolic disease, extreme intrauterine growth restriction, or unexplained critical illness. Conversely, WGS was not indicated for presentations, likely explained by nongenetic etiologies.

### Demographic and Clinical Settings Data

Of the NGC participants who provided full consent for medical record linkage, the final analytical cohort comprised successfully linked survivors with complete primary identifier data for deterministic linkage, resulting in 0 missing demographic variables for the analytic cohort. Participants’ ethnicities included African, East Asian, European, Finnish-European, South Asian, and other and were ascertained by parental report. Ethnicity was collected in the study to characterize the study population and assess potential differences in representation. Due to historical consent changes, raw data for unlinked individuals were omitted, and only final statistical comparisons were reported. To address potential selection and survivor biases, we evaluated cohort representativeness by comparing the baseline demographic and clinical settings of the final analytical cohort against the broader NGC and fully consented individuals. Further details regarding selection bias, cohort representativeness, and specific methodologies are included in the eMethods in Supplement 2.

### Clinical Phenotype Data

Data were obtained from electronic medical records via the NIHR BioResource. Unique Human Phenotype Ontology (HPO) terms (n = 1108) were identified, updated, and standardized via the Monarch Initiative.^33^ For targeted clinical analysis, the 3 most prevalent HPO terms across the cohort (hypotonia, seizures, and neurodevelopmental and developmental delay) were selected for individual evaluation. The standardized terms were mapped to level 3 parent terms, yielding 23 unique organ-level terms for uniform phenotypic representation for consistent feature comparison.

### DNA Variant Classification

Genomic data processing was performed on genetic variants. Variants were classified into pathogenic, likely pathogenic, and uncertain significance, using diagnostic criteria described previously.^28^ Individuals with any identified pathogenic or likely pathogenic variant or variants of uncertain significance were classified as genetically diagnosed, whereas remaining individuals were designated as genetically undiagnosed.

### Primary Care Records

Primary care records were accessed via the ECLIPSE Live platform.^27^ Individual general practices, which control primary care data in the UK, permitted access records for consenting participants. Deterministic linkage was performed within a secure data environment using pseudonymized patient identifiers, specifically NHS number, date of birth, and biological sex. The resulting longitudinal dataset captured health care utilization and associated costs spanning from January 2022 to June 2024. Costs are reported in pounds sterling (to convert to US dollars, multiply by 1.36). Methodologic details for health care cost estimation are provided in the eMethods in Supplement 2. Raw data were classified into 4 broad operational categories: hospital visits and admissions, prescriptions, pathology, and conditions (eTable 1 in Supplement 1).

The primary care dataset was processed into 36 distinct features. Highly sparse features within the pathology, prescription, and condition categories were aggregated into composite groups to mitigate matrix sparsity and optimize subsequent evaluation. A comprehensive data dictionary, with explicit feature engineering and derivation methods, is presented (eTable 2 in Supplement 1 and eMethods in Supplement 2).

### Statistical Analysis

#### Multiplicity Control

Group comparisons were performed using the Wilcoxon rank sum test. Because utilization data contained a high frequency of tied values, significance was calculated using an asymptotic normal approximation with a standard continuity correction. Effect sizes were quantified as Hodges-Lehmann median differences (95% CIs). A total of 288 statistical tests were conducted, encompassing 32 outcome features analyzed across 9 predefined clinical subgroups. Subgroups failing to meet a minimum sample size (n = 7 individuals or arm) were excluded. To strictly control the family-wise error rate, we adopted a conservative, fixed significance threshold of 2-sided *P* < .001. We analyzed data using R, version 4.5.0 (R Project for Statistical Computing).

#### Predictive Model Development and Feature Impact Analysis

**Figure 1.**
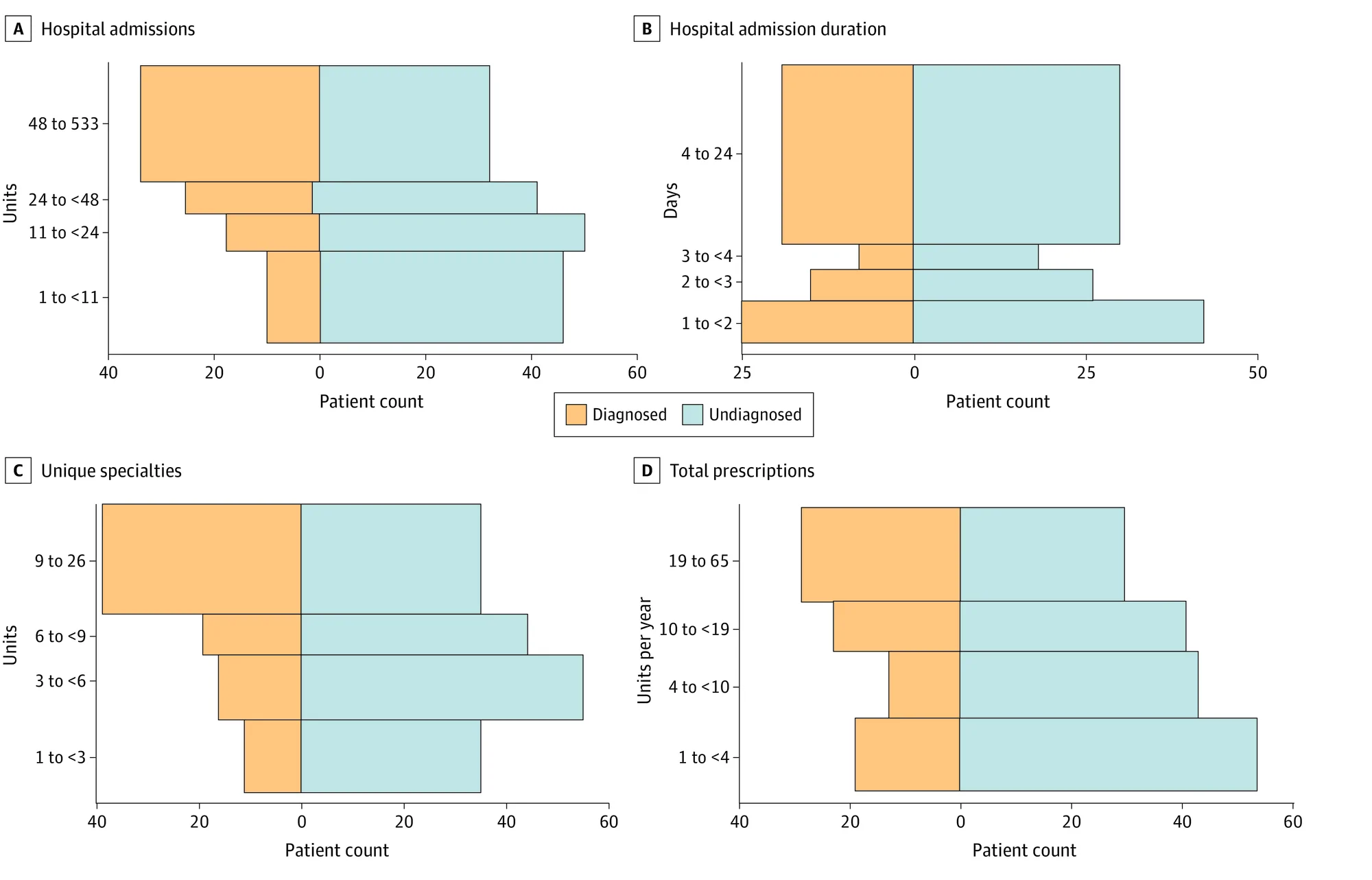
Histogram of Distribution of Statistically Significant Demographic and Primary Care Clinical Features Across the Study Cohort (N = 270), Stratified by Genetic Diagnostic Status Continuous features are discretized into 4 quantile-based bins to facilitate standardized multivariable comparisons. Horizontal bar lengths represent the absolute patient count within each bin along the x-axis (split symmetrically from the central origin at x = 0 for diagnosed vs undiagnosed groups). Vertical bars represent the absolute count of individuals within each bin, grouped by diagnostic status (diagnosed vs undiagnosed). To accommodate highly skewed distributions and improve visual resolution across disparate scales, the y-axes were transformed using a pseudo-log scale and display nonoverlapping mathematical interval ranges.

A binary classification framework was developed to predict genetic diagnostic outcomes by integrating demographic, clinical phenotype, and health care utilization features into a unified feature matrix (eTable 3 in Supplement 1). Clinical phenotypes were represented as a binary matrix using the 23 organ-level HPO terms derived during data processing. Categorical features were 1-hot encoded, while numeric features were discretized into 4 quantile-based intervals prior to 1-hot encoding. Histograms of demographic and primary care features, stratified by diagnostic group, with quantile-based bins were constructed (Figure 1 and eFigure 1 in Supplement 2).

Dimensionality reduction was executed using principal component analysis (PCA), followed by supervised linear discriminant analysis (LDA). PCA was conducted without centering or scaling to preserve the uniform Bernoulli variance structure and underlying matrix sparsity. Model stability was evaluated via 100 repeated random subsampling iterations (70% training and 30% validation split). To prevent data leakage, all model parameters were fitted exclusively on the training partition within each bootstrap loop. The optimal model configuration was selected based on the highest mean validation area under the receiver operating characteristic curve (AUROC).

Individual feature importance was evaluated using a leave-one-feature-out analysis conducted on the final PCA and LDA models. Comprehensive methodologic details are available in the eMethods in Supplement 2.

## Results

### Cohort Characteristics and Clinical Context

**Figure 2.**
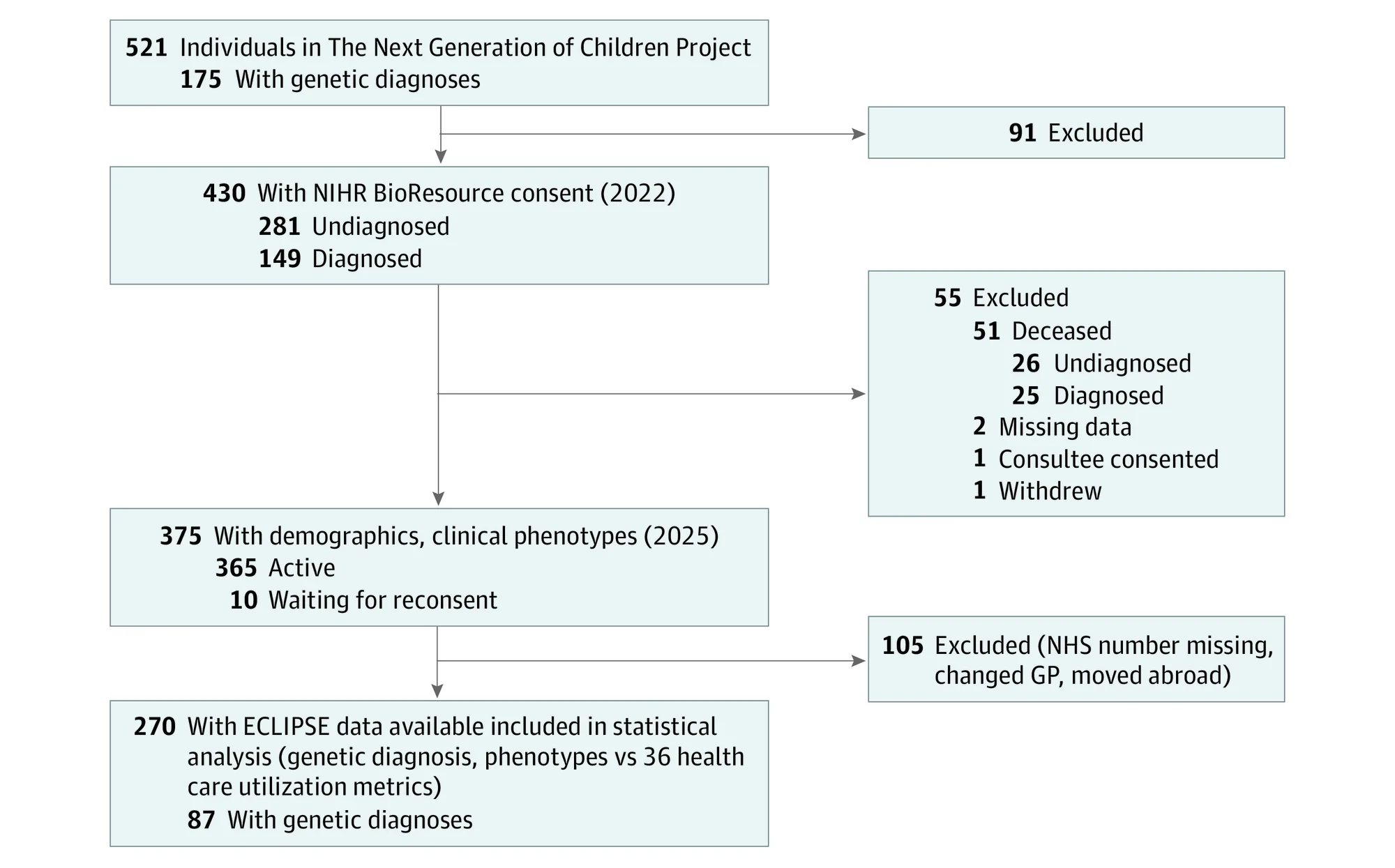
Flow Diagram Detailing Patient Progression From Assessment to Final Analysis ECLIPSE indicates Equality of Care Led Insights for Patient Safety & Engagement; GP, general practice; NHS, UK National Health Service; NIHR BioResource, UK National Institute for Health and Care Research Rare Disease BioResource.

Of 521 NCG recruits, 430 (82.5%) provided consent for medical record linkage (Figure 2). Within the consented group, mortality was significantly associated with genetic diagnostic status: 25 of 149 diagnosed individuals (16.8%) were deceased compared with 26 of 281 undiagnosed individuals (9.3%) (χ^2^ = 4.58; *P* = .032; Fisher exact, *P* = .03) (eTable 4 in Supplement 1). Successful linkage to ECLIPSE Live was performed for 270 of 375 living individuals (72%) (Figure 2). Unlinked individuals (n = 105) were excluded due to missing primary identifiers, withdrawn consent, patient mortality, or a change in primary care service. Postconsent exclusions were due to mortality (n = 51) and linkage failures or withdrawals (n = 109), resulting in a final analytical cohort of 270 individuals (mean [SD] age 8.65 [6.58-9.46] years; 121 females [44.8%] and 149 males [55.2%]).

Within the analytic cohort, 87 participants (32.2%) received a genetic diagnosis, similar to the full NGC cohort (34.0%) compared with 183 undiagnosed peers (67.8%). Among the 270 participants, 2 (0.7%) were African, 225 (83.3%) were European (83%), 1 (0.4%) was Finnish-European, 18 (6.7%) were South Asian, and 24 (8.9%) were of other ethnicities. Thirty percent of participants (n = 81) were in the Index of Multiple Deprivation (IMD) deciles 1 to 4 (lowest socioeconomic status group). Children were recruited from clinical settings including 94 (34.8%) from the NICU, 112 (41.5%) from neurodevelopmental clinics, and 64 (23.7%) from the PICU. The analytic cohort (recruited from December 2016 to August 2020) consisted of individuals from ages 5 to 22 years (analyzed in March 2025) with a slight male predominance and higher IMD than the full NCG cohort, but these differences were not statistically significant (eTables 5 and 6 in Supplement 1 and eFigure 2 in Supplement 2). While demographic testing showed no socioeconomic bias in cohort retention, our conclusions are strictly applicable to long-term survivors with continuous health care linkage.

### Health Care Utilization and Cost Patterns

**Figure 3.**
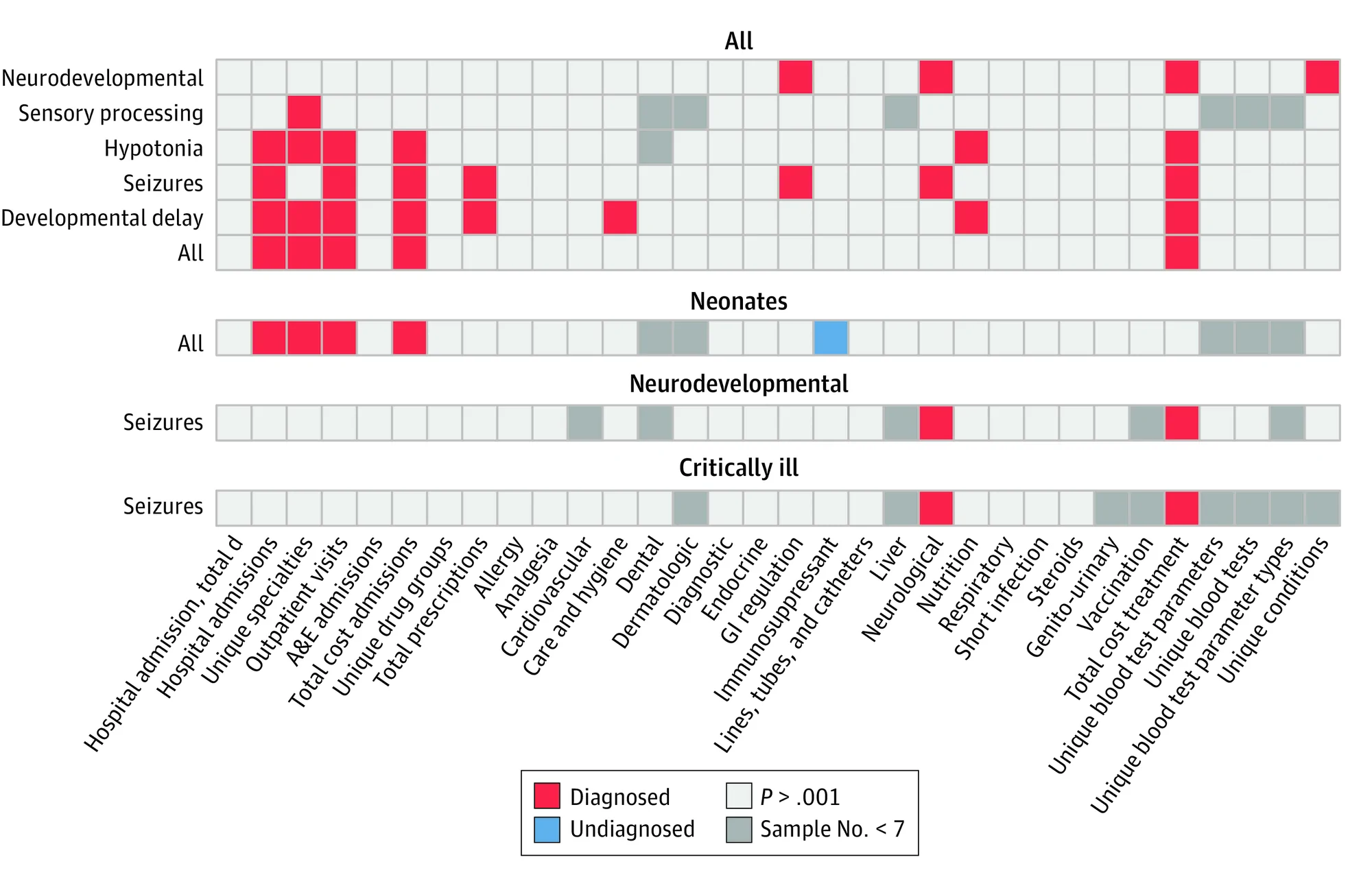
Heat Map of Increased Health Care Costs and Resource Utilization in Diagnosed Individuals Features are visually stratified based on whether they met the conservative significance threshold (*P* < .001 and n ≥ 7). For each condition and utilization metric, Wilcoxon rank sum tests were performed to compare diagnosed and undiagnosed individuals. The heatmaps use cells to indicate the group (diagnosed or undiagnosed) with the higher mean. A comprehensive master list of exact *P* values for all features shown, including those that did not meet the significance threshold, is detailed in eTable 8 in Supplement 1. A&E indicates accident and emergency department; GI, gastrointestinal.

To evaluate disparities in health care utilization and costs between genetically diagnosed and undiagnosed individuals, comparisons were conducted across clinical outcome features and predefined analytic subgroups using a conservative multiple-testing framework with family-wise error rate control (eTable 7 in Supplement 1). Diagnosed individuals consistently demonstrated significantly higher median (IQR) health care utilization and associated costs (hospital admissions: 37 [18-66] vs 22 [10-35]; *P* < .001) across primary comparisons) (Figure 3). A complete inventory of *P* values for all 288 analyzed features is provided in eTable 8 in Supplement 1. For comparisons meeting both the significance threshold (*P* < .001) and the minimum sample size requirement (n ≥ 7), the magnitude of the absolute difference is formally quantified in eTable 9 in Supplement 1.

**Figure 4.**
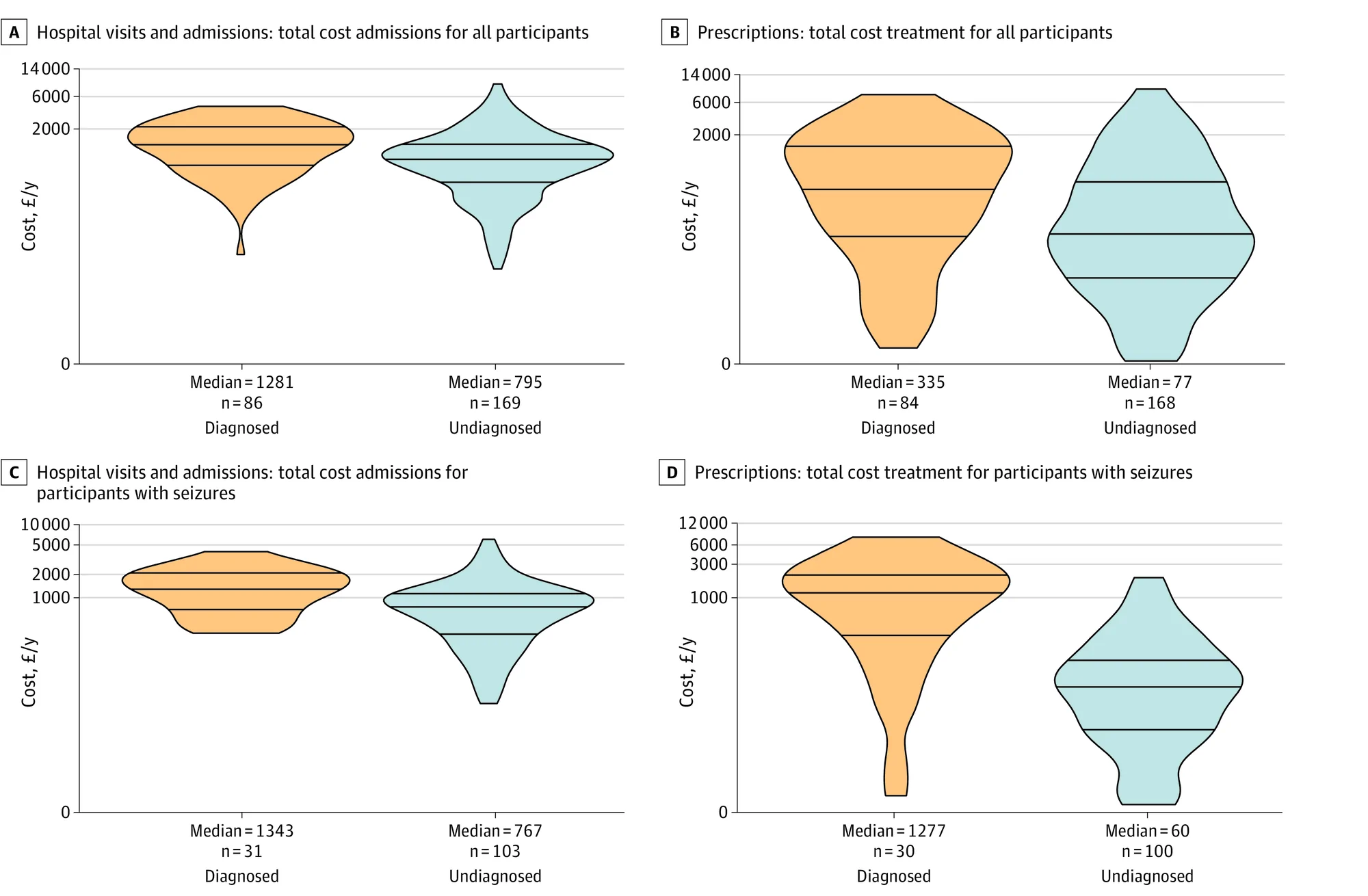
Violin Plots of Increased Health Care Costs and Resource Utilization in Diagnosed Individuals Each panel corresponds to a specific condition-utilization comparison, with violin plots contrasting diagnosed and undiagnosed groups (A, n = 255; B, n = 252; C, n = 134; D, n = 130). Horizontal lines within each violin indicate quartiles: first, 25th percentile; median, 50th percentile; and third, 75th percentile, bounding the IQRs. A pseudo-log scale to accommodate wide-ranging right-skewed cost distributions while preserving 0 values was used for the y-axis.

Diagnosed individuals experienced more median (IQR) hospital admissions (37 [18-66] vs 22 [10-35]), more unique specialties (8 [4-11] vs 5 [3-8]), more primary and secondary care outpatient visits each year (14 [7-26] vs 8 [4-13]) (eFigure 3A1-A3 in Supplement 2), and higher annual admission costs (£1281 [£652-£2291] vs £795 [£377-£1296]) (Figure 4A). These trends were particularly prominent in children with hypotonia (hospital admissions: 52 [29-78] vs 20 [8-33]) (eFigure 3C1 in Supplement 2), seizures (hospital admissions: 36 [20-66] vs 20 [8-33]; outpatient visits: 13 [8-26] vs 8 [3-12]) (eFigure 3D1 and D2 in Supplement 2), and developmental delay (hospital admissions: 36 [16-67] vs 20 [8-33]) (eFigure 3E1 in Supplement 2), although individuals with seizures did not show a difference in the number of specialties. Annual median (IQR) treatment costs were also higher for diagnosed participants (£335 [£71-£1400] vs £77 [£18-£430]) (Figure 4B), especially for genetic neurodevelopmental (£1280 [£507-£2529] vs £130 [£21-£354]) (eFigure 3F3 in Supplement 2) and seizure-related (£1277 [£320-£2271] vs £60 [£15-£143]) conditions (Figure 4D). Participants with diagnosed seizures had more median (IQR) prescriptions, particularly for neurological (48 [22-88] vs 0) and gastrointestinal (5 [1-18] vs 0 [0-2]) care (eFigure 3H1 and H2 in Supplement 2). Participants with developmental delays showed a median (IQR) increase in prescriptions for care and hygiene (1 [0-16] vs 0 [0-1]) (eg, catheters, feeding tubes, and positioning aids) and nutrition (4 [0-42] vs 0 [0-1]) (eFigure 3I1 and I2 in Supplement 2). All statistically significant differences across conditions and utilization metrics are presented in Figure 4 and eFigures 2-5 in Supplement 2).

The health care costs associated with a diagnosis, highlighting the higher total admission and treatment costs for diagnosed individuals, are presented in Figure 4C and D and eFigure 3D and H in Supplement 2). In children recruited from NICUs, those who were diagnosed had more median (IQR) readmissions (58 [38-94] vs 16 [6-33]), specialty involvement (12 [8-14] vs 5 [2-7]), and outpatient visits (21 [15-36] vs 6 [2-12]) and higher admission costs (£2092 [£1201-£2809] vs £716 [£546-£1247]) (eFigure 4A-D in Supplement 2). Median (IQR) differences specifically in neurodevelopmental (neurological prescriptions: 42 [22-64] vs 0 [0-3]) and PICU (total cost prescriptions: £1045 [£244-£1914] vs £38 [£14-£220]) settings are shown in eFigure 5A and B in Supplement 2, and those with critical care seizures (total cost prescriptions: £2438 [£757-£4734] vs £72 [£21-£301]) are shown in eFigure 6B in Supplement 2. Diagnosed individuals consistently exhibited significantly higher health care utilization and costs, including hospital admissions, outpatient visits, and prescription costs, with the most pronounced differences observed in children with seizures, hypotonia, and developmental delay.

### Predictive Model

To ascertain whether health care utilization patterns could inform the likelihood of obtaining a diagnosis via WGS, a model was trained on 70% of the cohort and validated on 30% (eFigure 7A in Supplement 2). LDA scores showed clear median (IQR) separation between diagnosed (0.62 [0.03 to 1.19]) and undiagnosed (−0.29 [−1.01 to 0.42]) individuals (*P* < .001), and the AUROC analysis (eFigure 7B and C in Supplement 2) resulted in an AUROC of 0.71 (sensitivity, 81%; specificity, 60%). The training AUROC was 0.78 (sensitivity, 79%; specificity, 64%), and full datasets had an AUROC of 0.76 (sensitivity, 79%; specificity, 63%).

**Figure 5.**
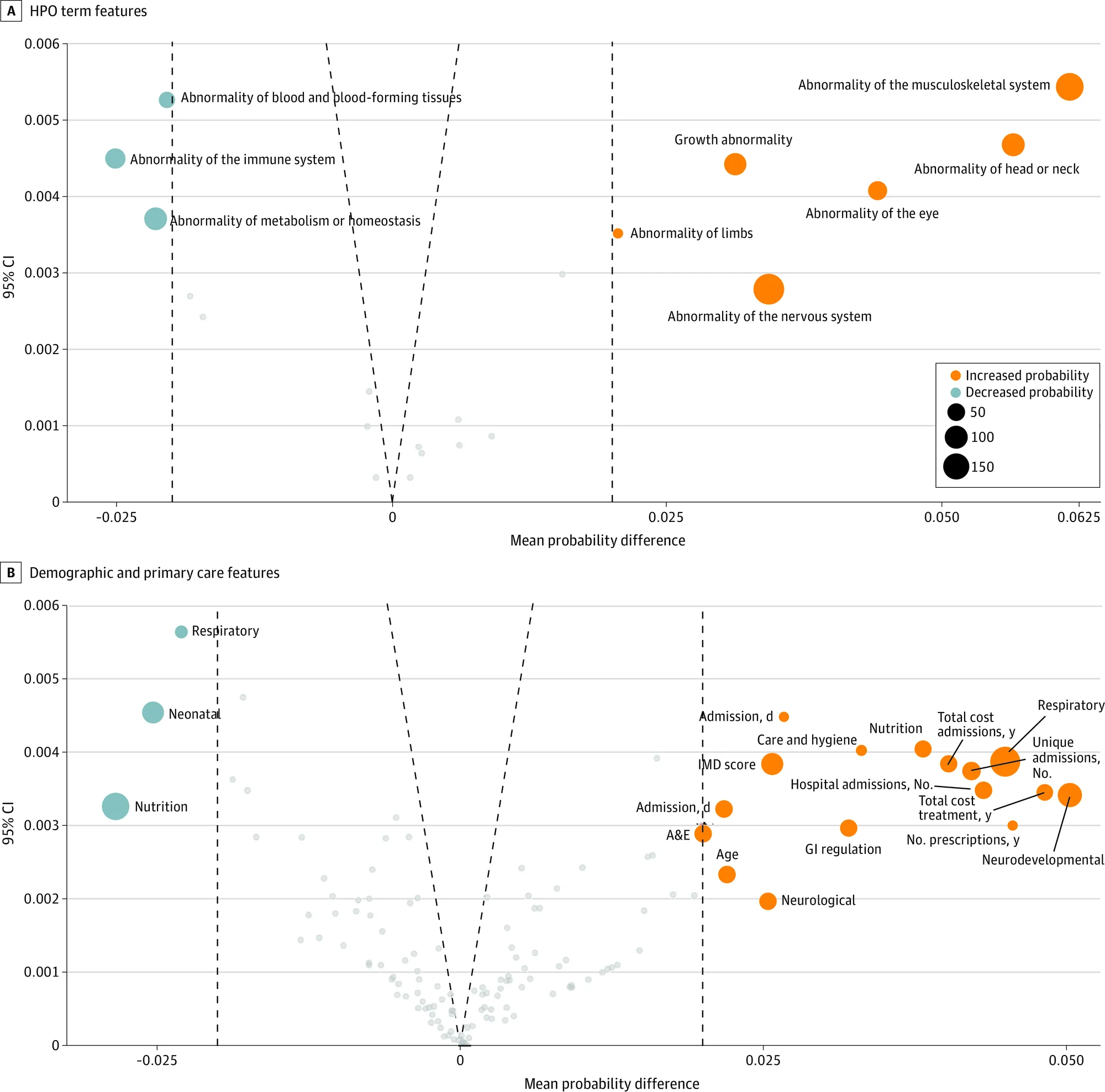
Volcano Plots Highlighting Human Phenotype Ontology (HPO) Terms and Demographics and Health Care Utilization Variable Intervals With Increased or Decreased Probability of a Genetic Diagnosis Features are labeled if the absolute mean probability difference is greater than 0.02 and exceeds the width of its 95% CI. The size of each data point is proportional to each variable frequency in the cohorts. A&E indicates accident and emergency department; GI, gastrointestinal; IMD, Index of Multiple Deprivation.

The feature impact analysis (Figure 5) identified key clinical indicators associated with diagnostic probability (eTable 10 in Supplement 1). Key predictors were older age, higher IMD decile, management in a neurodevelopmental unit, lower accident and emergency department attendance, higher hospital admissions, higher total admissions, and higher admission costs (from £1549 to £9201). Both low (1 to 2) and high (4 to 24) inpatient days were associated with increased likelihood of diagnosis. Treatment-related predictors included higher annual prescriptions; treatment costs; and prescriptions for nutrition, neurology, gastrointestinal regulation, and hygiene and care. Low respiratory prescriptions were associated with increased likelihood, while high respiratory prescribing, low nutrition prescriptions, and management in NICU were associated with a reduced likelihood. Six HPO systems (musculoskeletal, head and neck, growth, eye, limb, and nervous system) were key predictors of a diagnosis, whereas a lower likelihood was associated with abnormalities in blood, immune, and metabolic systems. We note that the LDA model, using PCA preprocessing, prioritized clarity but may have overlooked nonlinear effects and increased false positives. In sum, feature analysis of our model suggests that the likelihood of obtaining a positive diagnosis from pediatric WGS in the context of serious illness was associated with high health care utilization, specifically frequent hospital admissions and neurological prescriptions, whereas neonatal presentation and respiratory phenotypes were associated with a lower likelihood of obtaining a genetic diagnosis.

## Discussion

While several prior studies have indicated benefits and cost-savings of WGS in the short-term for children with severe illness, this cohort study investigated longer-term implications of a WGS diagnosis. We generated new insights by combining primary care records and genomic data of children with serious illness.

Genetic diagnosis via WGS was not associated with a reduction in overall long-term health care costs but rather was associated with more precise allocation of clinical resources tailored to individual patients. Individuals who received a genetic diagnosis used significantly more health care resources than undiagnosed peers including increased hospitalizations, outpatient visits, and condition-specific prescriptions for neurological, gastrointestinal, and nutritional management.

While associated HPO terms were equivalent between diagnosed and undiagnosed groups, children who received a diagnosis had enhanced use of specialist pathways and personalized treatment. The most pronounced differences in utilization patterns were observed among children initially diagnosed in intensive care settings (NICU or PICU), underscoring the clinical complexity of this population.

Such findings are important for informing clinical services. Our team’s previous work suggests that early detection could help prioritize faster management in the acute illness phase.^12,28^ We now show that diagnosed children can often access more specialized care after the acute phase. While this might not lower immediate costs, it optimizes long-term care for chronic neurodevelopmental disorders. Importantly, our results support the continued application of current NHS WGS eligibility criteria, which prioritize children with a high clinical suspicion of monogenic disorders, rather than expanding WGS inclusion to broader, nonspecific clinical categories.

None in our cohort were recipients of gene therapies, such as antisense oligonucleotides, attracting high annual costs. Our findings of higher health care utilization and cost are thus independent of considerations around advanced therapies, which is an important future consideration.

Health care utilization patterns were closely tied to phenotype-diagnosis associations. The leave-one-feature-out analysis showed that older age, higher socioeconomic status, and some aspects of prescribing and admissions were associated with a higher diagnostic yield. Notably, neonates in the undiagnosed group exhibited significantly higher immunosuppressant use. In the absence of a genetic diagnosis to inform genotype-directed therapy, this likely reflects a reliance on empiric immunomodulation for undifferentiated inflammatory states. It may also reflect complex acquired neonatal morbidities, such as chronic lung disease, underscoring the distinct therapeutic challenges inherent in managing neonates with unknown underlying etiologies.

### Limitations

This study had several limitations. We acknowledge that this study characterizes the inherent clinical complexity of the cohorts rather than any causal effect of a diagnostic intervention. A limitation of this observational design is the inability to decouple whether the diagnostic process itself was associated with increased costs via specialist interventions or whether these expenditures reflect the intrinsic baseline needs of managing genetic disease. Furthermore, these metrics do not clarify whether higher utilization reflects more targeted clinical management or is a by-product of frequent, sustained contact with the health care system. Consequently, these findings indicate high clinical complexity and systemic engagement rather than serving as direct proxies for care quality or therapeutic optimization.

Sample size constraints precluded a 3-way data split with an independent hold-out test set. Because such splits can compromise statistical power in smaller cohorts,^34,35^ we instead used a rigorous internal validation, in which the validation partition informed principal component selection within a nested loop. These results represent a stable internal estimate. However, subsequent evaluation in independent, multicenter external cohorts will be required to confirm the model’s accuracy and account for recruitment-era drift. Furthermore, while our models quantified clinical trajectories, they did not capture the lived experiences of navigating increased health care utilization. Qualitative research within this cohort has highlighted complex parental support needs in the postdiagnostic period,^36^ as well as varying parent perceptions of how genomic results may alter medical care and support availability.^37^ Future research should integrate these qualitative experiences with utilization metrics to comprehensively evaluate the broader impacts of genomic diagnosis.

Further limitations include a relatively small analytical dataset. We were unable to include participants who did not consent for linkage studies, who had died, and who were not registered with a general practice with available linked data. The attrition between consent and final linkage introduced a potential source of selection bias. Because diagnostic status and clinical acuity are often correlated with early mortality, this survivor-only cohort may underrepresent the highest-intensity health care utilization associated with the most severe genetic presentations (eTable 11 in Supplement 1). Consequently, our findings may provide a conservative estimate of the true utilization gap. Conversely, an early diagnosis may facilitate transition to palliative care, which substantially reduces subsequent health care costs; thus analysis may also underestimate the total economic impact of early diagnosis. While statistical comparisons confirmed that the final cohort remained representative of the original study population in terms of baseline demographics (eTables 5 and 6 in Supplement 1 and eFigure 2 in Supplement 2), the potential for survivor bias must be considered.

Furthermore, we acknowledge the potential for confounding by phenotypic severity. Children with severe or multisystemic presentations are more likely to both receive a pathogenic diagnosis and require intensive, sustained medical follow-up. Since this study did not use propensity-based matching or standardized clinical severity scores, the observed utilization gap reflects a combination of the diagnostic state and the intrinsic severity of the underlying condition, characterizing the aggregate burden rather than isolating the independent economic impact of the diagnosis itself. In addition, the up-front costs of WGS were not available for inclusion. However, this aligns with using accumulated cost as a standardized proxy for systemic health care utilization rather than conducting a formal cost-effectiveness evaluation. Additionally, while our models adjusted for baseline socioeconomic status using IMD scores, our dataset lacked granular family-level covariates or geographic parameters. Unmeasured confounders, such as regional health care accessibility and parental health status, could independently modulate health care utilization.

## Conclusions

The findings of this cohort study show feasibility of linkage of genetic data to community or general practice medical records and mapping of health care utilization needs at scale for children with diagnosed rare genetic conditions. Children with conditions diagnosed via WGS had significantly higher costs for care over time than those without an identified genetic diagnosis. While these findings do not measure the effect of WGS as a clinical intervention, they provide a longitudinal characterization of health care utilization patterns and highlight the profound clinical complexity and sustained resource requirements inherent in monogenic disorders. The findings suggest that a WGS diagnosis, rather than reducing overall costs, may facilitate integration of specialist care, helping align health care resources with individual needs. Future work should validate these findings in different populations. Long-term strategies should focus on predicting care intensity and treatment needs, with cost evaluations that include primary, secondary, and social care costs.

## Notes

Supplement 1. **eTable 1. **Primary Care Features Used in the Analysis, Including Outpatient Visits, Prescriptions, Pathology, and Conditions **eTable 2.** Data Dictionary for ECLIPSE-Provided Variables and Their Transformation for Use in This Study **eTable 3.** Case-Level Feature Matrix Used for Predictive Modeling **eTable 4.** Attrition Analysis and Differential Mortality by Diagnostic Status **eTable 5.** Distribution of Age, Gender, Ethnicity, IMD Score, Clinical Setting, and Genetic Diagnosis **eTable 6.** Assessment of Cohort Representativeness Based on Consent and Linkage Status **eTable 7. **Distribution of Cases Across the Full Cohort and Within Each Hospital Unit **eTable 8.** Statistical Significance of Differences in Health Care Utilization Between Diagnosed and Undiagnosed Cases **eTable 9.** Effect Size Estimates for Statistically Significant Health Care Utilization Differences **eTable 10.** Numerical Values of Feature Impacts on Diagnostic Probability **eTable 11.** Comparison of Diagnostic Yield by Clinical Acuity Setting

Supplement 2.
eMethods
**eFigure 1. **Histogram of Demographic and Primary Care Features Across 270 Cases, Stratified by Diagnostic Group **eFigure 2.** Assessment of Cohort Representativeness and Selection Bias Across Age and Socioeconomic Deprivation Metrics **eFigure 3.** Violin Plot Visualization of Health Care Utilization: Hospital Visits and Admissions, Prescriptions, and Unique Conditions **eFigure 4.** Violin Plot Visualization of Health Care Utilization: Hospital Visits and Admissions and Prescriptions **eFigure 5.** Violin Plot Visualization of Health Care Utilization: Neurodevelopmental Prescriptions **eFigure 6.** Violin Plot Visualization of Health Care Utilization: Critically Ill Prescriptions **eFigure 7.** Predictive Modeling of Genetic Diagnosis

Supplement 3.
Data Sharing Statement
