# Supplementary material for: Diagnosis Through Whole Genome Sequencing and Care Utilization in Children With Severe Illness
Source: JAMA Netw Open. 2026 Sep 17;9(9):e2634372. doi: 10.1001/jamanetworkopen.2026.34372 (PMC13586901; doi:10.1001/jamanetworkopen.2026.34372)
Supplement: Supplementary file 1 [file jamanetwopen-e2634372-s002.pdf]

Supplemental Online Content

Dias JML, More RP, Butler D, et al. Long-term secondary care utilization in a survivor cohort of children with serious illness. *JAMA Netw Open*. 2026;9(9):e2634372.
doi:10.1001/jamanetworkopen.2026.34372

eMethods
eFigure 1. Histogram of Demographic and Primary Care Features Across 270 Cases, Stratified by Diagnostic Group
eFigure 2. Assessment of Cohort Representativeness and Selection Bias Across Age and Socioeconomic Deprivation Metrics
eFigure 3. Violin Plot Visualization of Health Care Utilization: Hospital Visits and Admissions, Prescriptions, and Unique Conditions
eFigure 4. Violin Plot Visualization of Health Care Utilization: Hospital Visits and Admissions and Prescriptions
eFigure 5. Violin Plot Visualization of Health Care Utilization: Neurodevelopmental Prescriptions
eFigure 6. Violin Plot Visualization of Health Care Utilization: Critically Ill Prescriptions
eFigure 7. Predictive Modeling of Genetic Diagnosis

This supplemental material has been provided by the authors to give readers additional information about their work.

© 2026 Dias JML et al. *JAMA Network Open*.

eMethods

Assessment of Cohort Representativeness and Selection Bias

To assess potential selection bias associated with consent to data linkage and successful linkage to ECLIPSE, we compared age, biological sex, and Index of Multiple Deprivation (IMD) scores between cases that consented to linkage (n = 375) versus those that did not (n = 146), and cases successfully linked to ECLIPSE (n = 270) versus those that were not linked (n = 105). Age and IMD score were compared using Wilcoxon rank-sum tests implemented in R using wilcox.test(x, y, conf.int = TRUE, exact = FALSE), providing asymptotic p-values with continuity correction and Hodges-Lehmann median differences with 95% confidence intervals. Biological sex distributions were compared using chi-squared tests (chisq.test) with Fisher's exact tests (fisher.test) computed as a confirmatory measure.

Association Between Clinical Acuity and Diagnostic Yield

Association Between Clinical Acuity and Diagnostic Status

An additional analysis was performed to examine the relationship between clinical acuity setting and genetic diagnostic status within the linked study cohort. Only cases successfully linked to ECLIPSE were included in this analysis.

Patients were grouped according to their primary clinical context into two categories: High Acuity (ICU), comprising Neonates and Critically Ill cases, and Lower Acuity (Neurodevelopmental), comprising Neurodevelopment cases.

A 2×2 contingency table was constructed to compare the distribution of diagnosed and undiagnosed cases across acuity groups. The association between clinical acuity and diagnostic status was evaluated using Fisher's exact test implemented in R with fisher.test, which was used as the primary test. For completeness, a Pearson chi-squared test was also computed using chisq.test.

Attrition analysis and differential mortality by diagnostic status

To characterize potential selection and survivor biases, we examined attrition and mortality patterns stratified by diagnostic status within the subset of 430 individuals who provided initial record-linkage consent out of the 521 total enrolled cases. Diagnostic status and long-term outcomes could not be verified for individuals who did not provide consent and were therefore excluded from this analysis.

This assessment could not be performed because, while no deceased cases were linked, the number of deceased consented individuals contributing to linkage attrition was not available.

A 2 × 2 contingency table was constructed comparing mortality status (deceased vs alive) between diagnosed and undiagnosed individuals with available outcome data. Consistent with the statistical approach applied throughout the manuscript, Fisher's exact test was used as the primary test to evaluate the association between diagnostic status and early mortality, given the binary outcome structure. Pearson's chi-squared test with Yates' continuity correction was additionally computed as a complementary measure.

Healthcare Cost Estimation: The cost of care per patient was estimated using routinely collected prescribing and hospital activity data and should be interpreted as an indicative, rather than fully validated, economic measure. Medication costs were derived from prescriptions issued to each patient during the study period. Cost estimates were based on standard prescribing cost references and assumed one dispensed pack per prescription item unless more detailed dispensing information was available. As such, the prescribing cost calculations may not fully reflect actual dispensing quantities, adherence, rebates, or local procurement arrangements. Hospital admission costs were estimated using the relevant NHS Secondary Uses Service (SUS) Payment by Results (PbR) tariff values associated with recorded admission activity. Where no directly matched tariff was available, costs may have been unavailable or estimated using the closest applicable reference category. These figures represent unverified indicative tariff-based costs and do not constitute audited financial expenditure or patient-level costing data. Accordingly, all reported cost figures should be interpreted as approximate comparative estimates intended to support population-level evaluation rather than precise accounting measures.

Statistical Analysis and Multiplicity Control

All group comparisons of longitudinal healthcare utilization counts and costs were performed using two-tailed Wilcoxon rank-sum tests. p-values were computed using the asymptotic normal approximation with a continuity correction, with exact probability calculations disabled. To avoid unstable estimates arising from small sample sizes, inferential testing was restricted to predefined clinical and phenotypic subgroups containing a minimum of seven individuals per diagnostic arm. Under this rule, subsets failing to meet this threshold (specifically, the cardiorespiratory and endocrine/immunological subgroups) were systematically excluded from inferential testing. Out of 36 healthcare utilization features initially collected, four were used exclusively to index clinical condition cohorts and were omitted from outcome testing. The remaining 32 utilization features were evaluated across nine valid analytical subgroups, comprising combinations of cohort membership (overall cohort, Neonates, Neurodevelopment, and Critically Ill) and clinical stratifications, yielding a maximum of 288 statistical comparisons across the study.

To limit false-positive findings across these comparisons, we implemented a stringent multiplicity control strategy focused on Family-Wise Error Rate (FWER) control rather than False Discovery Rate (FDR) control. Multiplicity was addressed by treating the 32 utilization features evaluated within any single subgroup as a distinct family of hypotheses. To control the within-family probability of at least one false positive α=0.05, a standard Bonferroni correction yields an operational threshold of p=0.05/32, which is approximately 0.00156. Rather than applying this exact fraction, we adopted a more

© 2026 Dias JML et al. *JAMA Network Open*.

conservative, fixed significance threshold of p<0.001. This strict threshold ensures that the FWER is maintained well below 0.05 for each subgroup, prioritizing specificity over sensitivity given the observational design and correlated structure of healthcare utilization outcomes. Importantly, under this framework, our chosen p-value threshold must be interpreted as a conservative, Bonferroni-equivalent adjustment for FWER control, rather than as an approximation to a Benjamini-Hochberg false discovery rate. Only contrasts simultaneously meeting this p<0.001 threshold and the minimum sample size of seven observations per diagnostic arm were recognized as statistically significant.

Because statistical significance alone does not convey the magnitude or clinical relevance of utilization differences, particularly in observational frameworks where minor effects can achieve nominal significance, hypothesis testing was paired with formal effect size estimation. For all contrasts meeting the predefined significance criteria, we calculated non-parametric Hodges-Lehmann estimators alongside corresponding 95% confidence intervals. The Hodges-Lehmann estimator provides a robust measure of median differences that is highly suited for skewed, heteroscedastic healthcare utilization counts and costs. Confidence intervals were derived using rank-based methods equivalent to those implemented in standard statistical software via the wilcox.test(conf.int = TRUE). All statistically significant contrasts reported in the main text figures and tables are accompanied by these effect size metrics within the Supplementary Information to facilitate direct interpretation of absolute clinical and health-system impacts.

Predictive model pipeline

The models were built using all available HPO terms, demographics and primary care records (named as features) from this study. Obsolete HPO terms were manually updated using the Monarch Initiative website (https://monarchinitiative.org/). A binary classification method was developed to predict genetic diagnostic outcomes from patient phenotypic data and primary care records. This method integrates Principal Component Analysis (PCA) for data dimensionality reduction with a supervised Linear Discriminant Analysis (LDA) classification model. The complete analysis was implemented in R (v4.5.0).

Step 1: Prepare Input Data

The model requires four data inputs: a binary matrix of HPO terms, demographics and primary care records (transformed features: model input matrix) and a binary vector indicating diagnostic status (model output vector).

- *Diagnostic Status Vector*: Cases were categorized into a positive (diagnostic) group and a negative (non-diagnostic) group.
  - *Positive Group*: Included cases with ‘Reported’ results.
  - *Negative Group*: included cases with ‘Non-diagnostic’ results.
- *Binary HPO Matrix*: All HPO terms were curated using the ‘ontologyIndex’ R package to ensure consistency, with obsolete terms updated to their current valid equivalents. The curated set of terms was retained for downstream analysis. HPO terms more specific than level 3 in the ontology's hierarchy were replaced by their corresponding ancestor at level 3. Terms at or broader than level 3 were retained. This process resulted in a binary matrix representing the presence or absence of normalized HPO terms for each case (rows) and HPO term (columns).
- *Demographics*: Biological sex and ethnicity were converted to binary vectors. Age was divided into three quantile-based intervals using breakpoints at 6 and 11 years-old and IMD scores were divided into five quantile-based intervals using breakpoints at the 2, 4, 6 and 8. This transformation allowed to convert these features into binary matrices.
- *Primary care*: Numeric features were divided into four quantile-based intervals using breakpoints at the 0th, 25th, 50th, 75th, and 100th percentiles, rounded up for interpretability. Features with four or fewer unique values were one-hot encoded. Missing values were encoded as zeros. This transformation allowed to convert these features into binary matrices.

Step 2: Optimize Number of Principal Components (PCs) via bootstrapping

To find the optimal number of PCs for the model, a bootstrapping procedure is performed by iterating over a candidate range of PC counts. For each candidate PC count, 100 bootstrapped iterations are run as follows:

- *Sample Data*: 70% of the data is sampled for training, ensuring a balanced class distribution is maintained.
- *Apply PCA*: PCA is performed on the binary feature matrix of the training sample using the ‘prcomp’ function, without centering or scaling the data.
- *Train LDA Model*: An LDA model is trained on the PCA-transformed training data. The model is built to generate a classification probability vector classifying cases into their predefined diagnostic groups using the 'lda' function from the 'MASS' package (v2.2.2) with default parameters.
  OR
- *Predict and Evaluate:* The complete dataset is projected into the PCA space derived from the training sample. The trained LDA model is then used to predict the genetic diagnostic status for the full dataset.
- *Store Results:* The Area Under the Curve (AUC) for the iteration is calculated from the Receiver Operating Characteristic (ROC) curve using the ‘roc’ function from the ‘pROC’ package (v1.18.5). The AUC and the PCA + LDA model from the iteration are stored.

© 2026 Dias JML et al. *JAMA Network Open*.

Step 3: Select Optimal Number of PCs
The best PC count is identified by calculating the average AUC across the 100 iterations for each tested PC count. The number of PCs that yields the highest average AUC is selected as the optimal number.

Step 4: Select Final Model
From the 100 models trained using the optimal number of PCs (determined in Step 3), the single model whose AUC is closest to the median AUC of that set is selected as the final model.

Step 5: Determine Best Probability Threshold
Using the predictions from the final model selected in Step 4, the optimal probability threshold is identified. This threshold is the point that maximizes the sum of Sensitivity and Specificity.

Step 6: Final Output
This pipeline generates the final, optimized components for the PCA + LDA genetic diagnostic model:
- Optimal number of PCs
- Selected PCA+LDA model
- LDA scores
- Optimal classification probability threshold

Feature Contribution Analysis Pipeline
The contribution of input features to the model's predictions was assessed to determine the influence of individual clinical features. This protocol describes the specific methodology for calculating this contribution.

Step 1: Define Input Data
The following data and a pre-trained model are required for the analysis:
- *Normalised HPO terms, demographic data and primary care records matrix*: A binary matrix, where rows represent individual cases and columns represent each of the input features. The values within the matrix indicate the presence or absence of a specific feature for each case.
- *Trained PCA + LDA model*: The combined PCA and LDA model trained for classification.

Step 2: Initialize Change Matrix
This step creates a matrix to store the results of the analysis.
- A change_matrix is generated with dimensions corresponding to the input data.
- All values in this matrix are initialized to 0. It will store the calculated difference in prediction probabilities.

Step 3: Iterative Contribution Calculation
This step systematically calculates the change in the model's prediction probability by removing one feature at a time for each case.
- *Outer Loop*: For each feature j:
- *Inner Loop*: For each case i:
  - Check for term presence: If feature[i, j] == 1, proceed with the following steps. Otherwise, skip to the next case.
  - *Get original classification probability*:
    - Apply the PCA + LDA model to the original feature vector for case i (feature[i, :]).
    - The resulting prediction probability is stored as the original probability (oProb_matrix[i, j]).
  - *Create modified case*:
    - Create a temporary copy of the case's feature vector, feature_modified[i, :].
    - In this copy, set the value for term j to 0 (feature_modified[i, j] = 0).
  - *Get Modified Classification Probability*:
    - Apply the PCA + LDA model to the feature_modified [i, :] vector.
    - The resulting prediction probability is stored as the modified probability (mProb_matrix[i, j]).
  - *Calculate classification probability change*:
    - Store the difference between the original and modified probabilities in change_matrix[i, j] = oProb_matrix[i, j] - mProb_matrix[i, j].

Step 4: Generate Final Output
The result of this pipeline is an aggregated score for each feature, representing its average influence on the model's output.
- For each feature j, calculate the average contribution by taking the mean of all values in change_matrix[:, j] where the term was originally present (feature[i, j] == 1).
- Calculate the respective 95% confidence intervals for each average contribution score.

© 2026 Dias JML et al. *JAMA Network Open*.

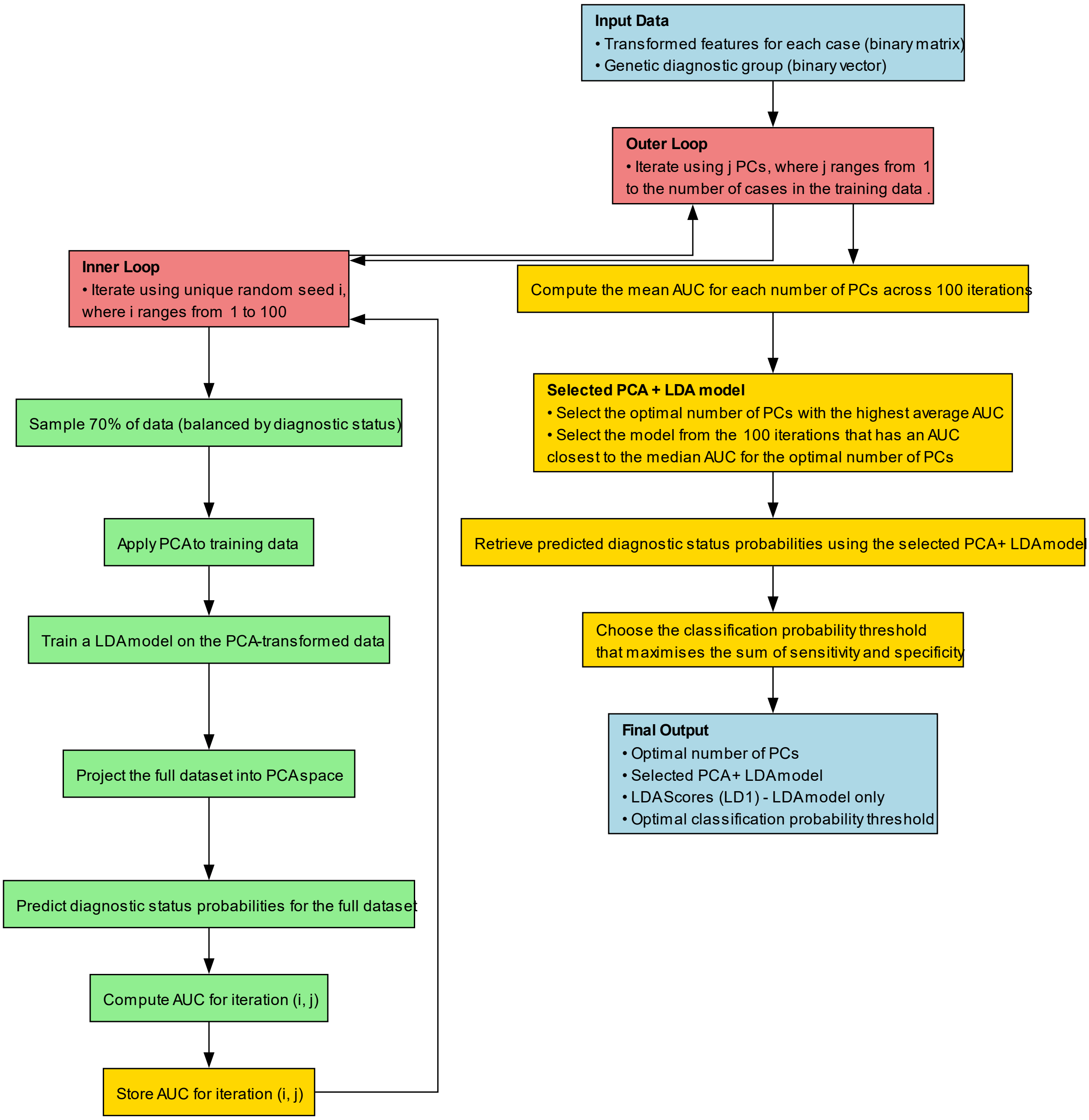

This diagram illustrates the process of predicting diagnostic status from the transformed features, using PCA for dimensionality reduction and LDA for classification. The methodology employs a nested loop structure to determine the optimal number of principal components and select a final, robust model. Each step is visually distinguished by colour: blue boxes represent the initial input data and final outputs; red boxes indicate the outer loop (iterating through the number of PCs) and the inner loop (100 bootstrapped iterations); green boxes show the core computational tasks; and yellow boxes mark the supporting tasks for result aggregation and model selection.

© 2026 Dias JML et al. *JAMA Network Open*.

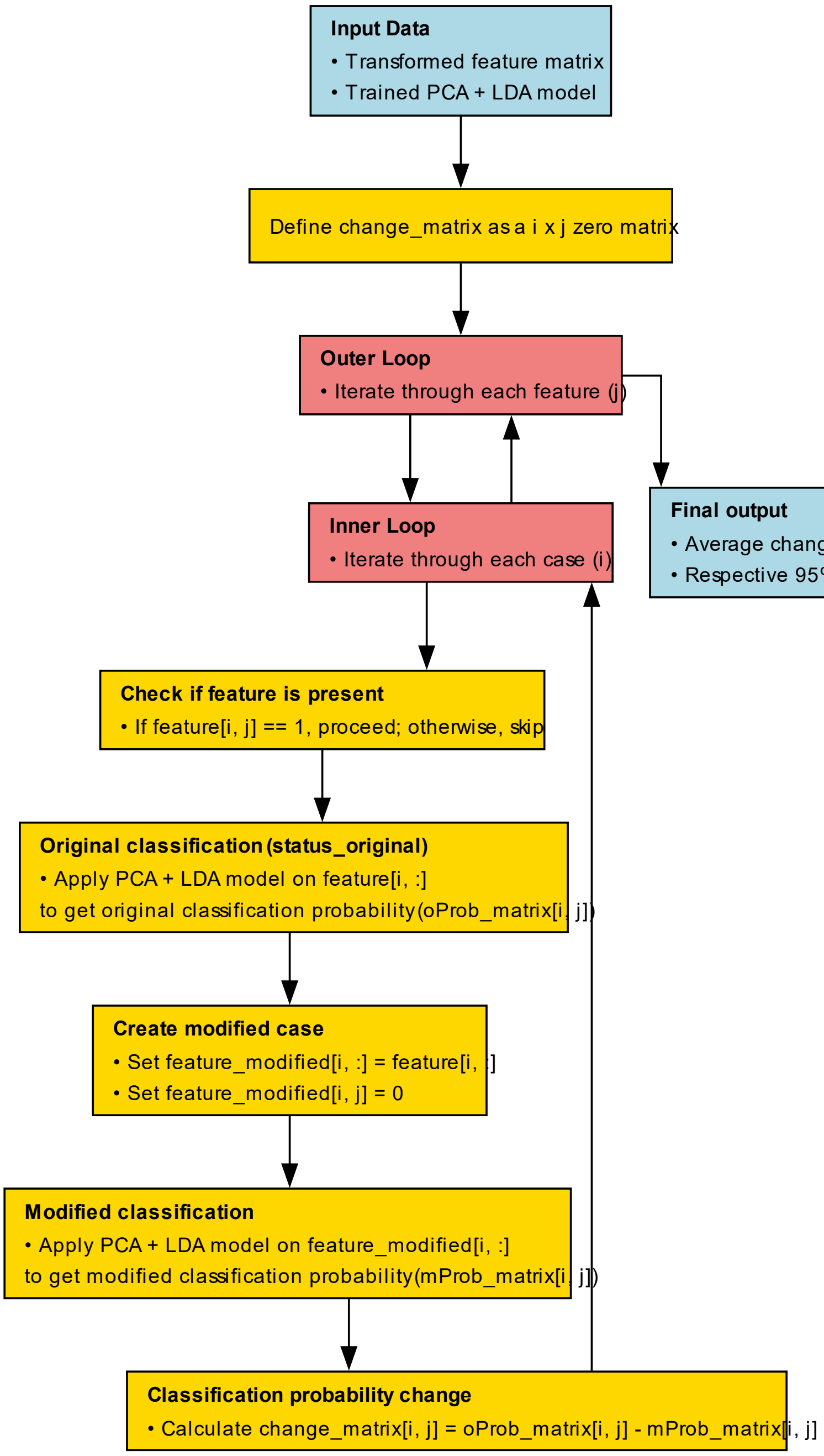

This flowchart details the process of quantifying the impact of removing each transformed feature on the classification probability generated by the selected PCA + LDA model. The methodology uses a nested loop to iterate through each term and each case. For cases where a term is present, the change in classification probability upon its removal is calculated. The final output is the average of these changes for each term, providing a measure of its overall diagnostic importance. Each step is visually distinguished by colour: blue boxes represent input and output, red boxes indicate loops, and yellow boxes show the core computations and supporting tasks.

© 2026 Dias JML et al. *JAMA Network Open*.

eFigure 1. Histogram of Demographic and Primary Care Features Across 270 Cases, Stratified by Diagnostic Group

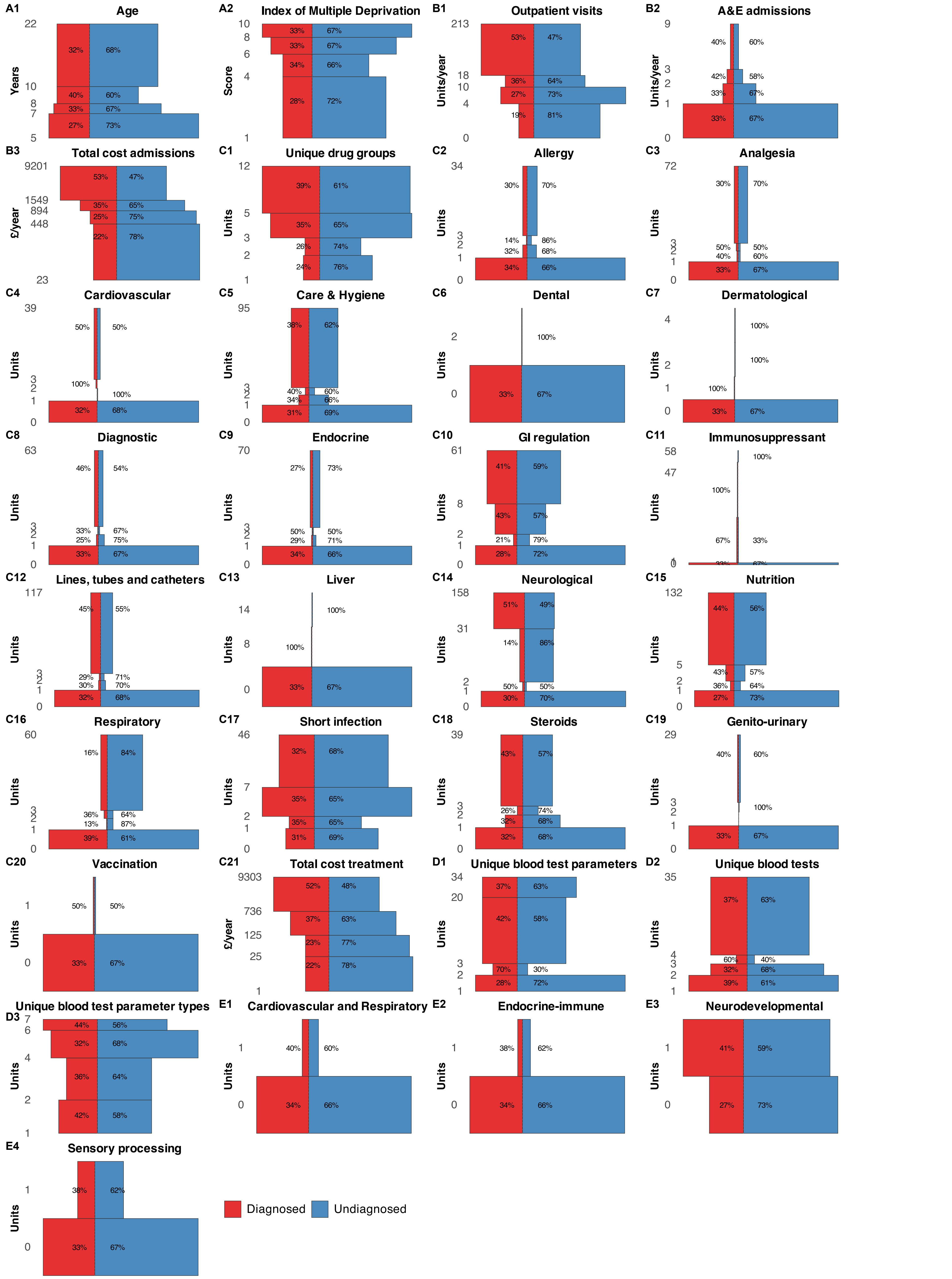

Each panel groups features from the same category: (A) Demographic characteristics; (B) Hospital visits & admissions; (C) Prescriptions; (D) Pathology and (E) Clinical conditions. Each feature is discretized into four quantile-based bins to enable standardized comparisons across variables. Bars indicate the absolute number of individuals per bin, grouped by diagnostic status (diagnosed vs. undiagnosed). To enhance visibility for features with high or skewed values, the y-axis is scaled using log1p.

© 2026 Dias JML et al. *JAMA Network Open*.

eFigure 2. Assessment of Cohort Representativeness and Selection Bias Across Age and Socioeconomic Deprivation Metrics

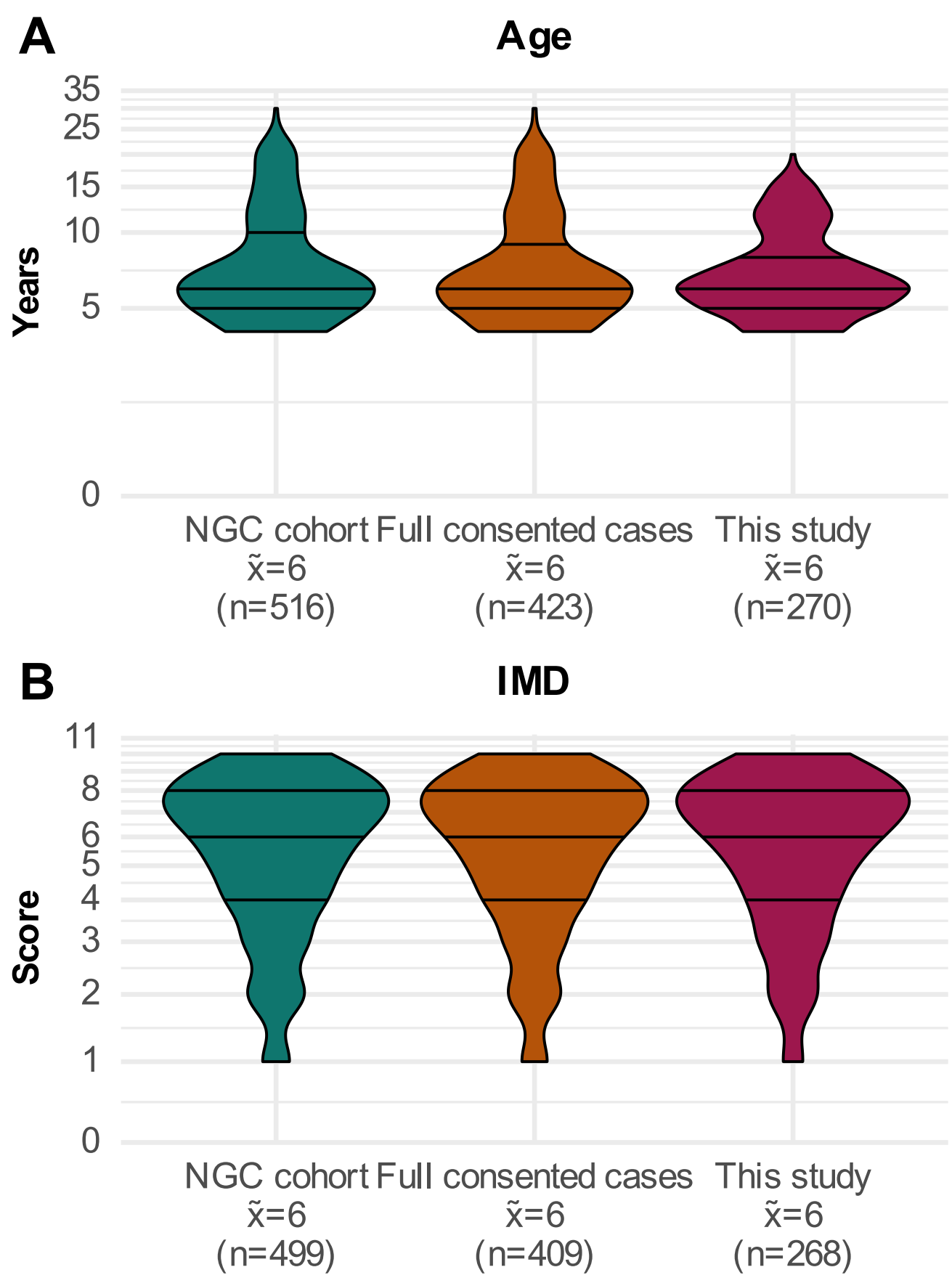

Violin plots illustrate the distribution of continuous baseline characteristics to evaluate potential biases across the data linkage for (A) Age distribution and (B) IMD scores. Each plot displays three distinct cohorts side-by-side: the NGC cohort (n=521), full consented cases (n=430), and this study (n=270). Internal horizontal lines denote the median and interquartile range (IQR) boundaries. The specific sample size and median value for each group are annotated directly below the x-axis. The y-axis uses a pseudo-log scale to accommodate wide-ranging values. Comprehensive group-comparison statistical outputs (Wilcoxon rank-sum test p-values, Hodges-Lehmann effect sizes, and confidence intervals) are provided in eTable 6.

© 2026 Dias JML et al. *JAMA Network Open*.

eFigure 3. Violin Plot Visualization of Health Care Utilization: Hospital Visits and Admissions, Prescriptions, and Unique Conditions

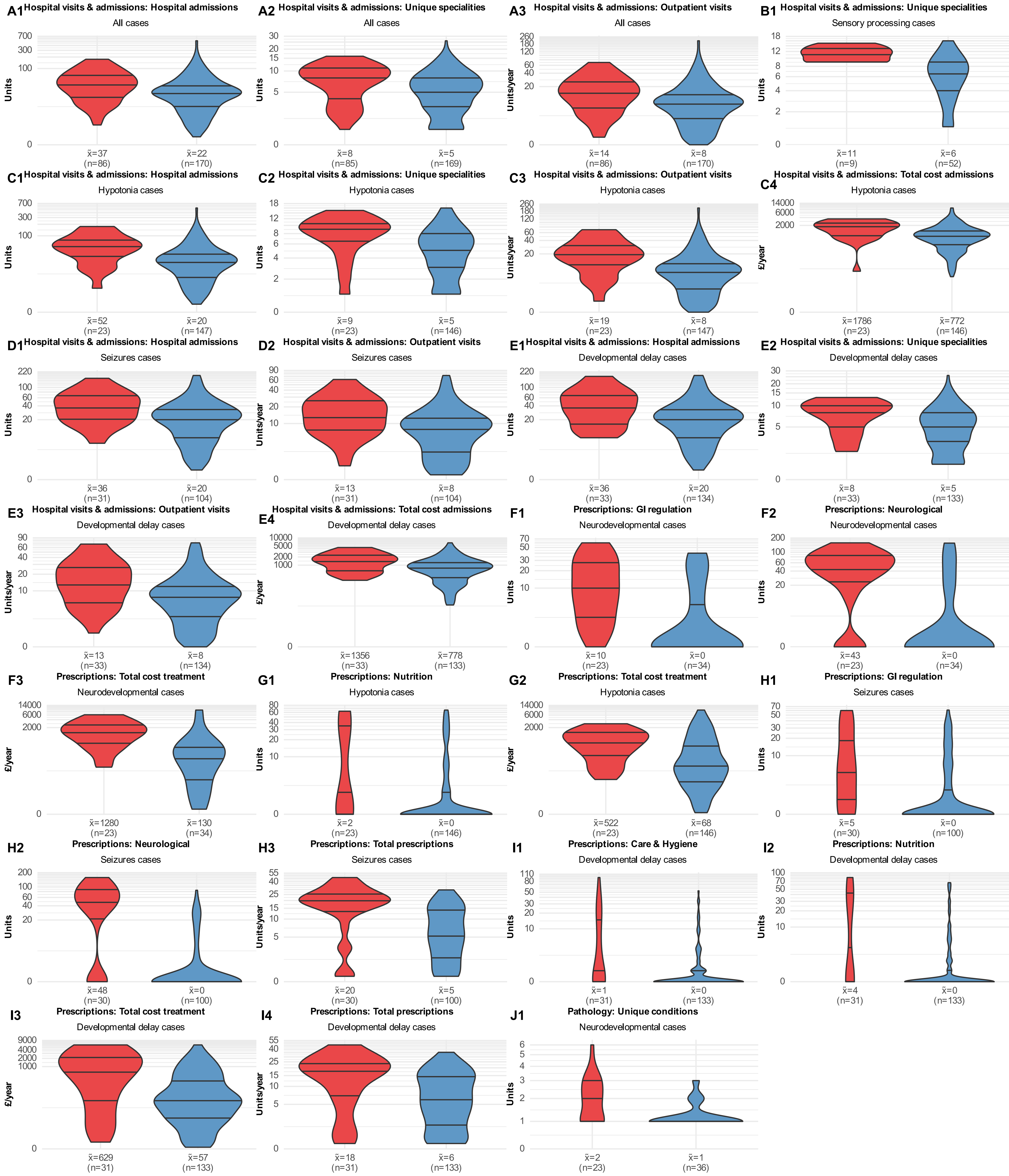

A-E: Hospital visits & admissions; F-I: Prescriptions; J: Conditions, highlighting statistically significant differences ($n \geq 7$; $p < 0.001$) between diagnosed and undiagnosed groups across the cohort (A: All) and multiple clinical conditions (B: Sensory processing; C, G: Hypotonia; D, H: Seizures; E, I: Developmental delay; F, J: Developmental). Each panel corresponds to a specific condition-utilization comparison, with violin plots contrasting diagnosed (red) and undiagnosed (red) groups. The y-axis uses a pseudo-log scale to accommodate wide-ranging values. Median, interquartile range (IQR), and sample size for each group are annotated below the respective plots.

© 2026 Dias JML et al. *JAMA Network Open*.

eFigure 4. Violin Plot Visualization of Health Care Utilization: Hospital Visits and Admissions and Prescriptions

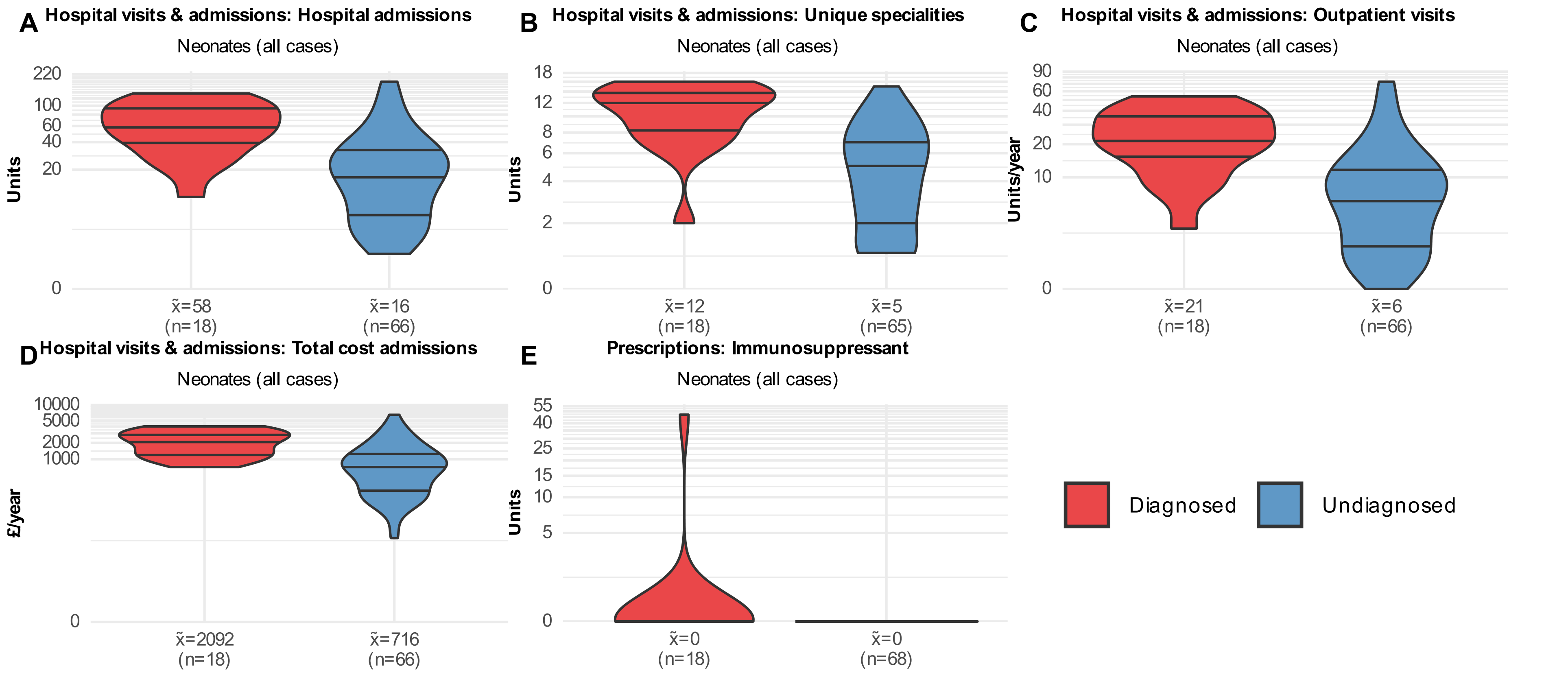

A-D: Hospital visits & admissions; E: Prescriptions, highlighting statistically significant differences (n ≥ 7; p < 0.001) between diagnosed and undiagnosed groups across the Neonates cases. Each panel corresponds to a specific condition-utilization comparison, with violin plots contrasting diagnosed (red) and undiagnosed (red) groups. The y-axis uses a pseudo-log scale to accommodate wide-ranging values. Median, interquartile range (IQR), and sample size for each group are annotated below the respective plots.

© 2026 Dias JML et al. *JAMA Network Open*.

eFigure 5. Violin Plot Visualization of Health Care Utilization: Neurodevelopmental Prescriptions

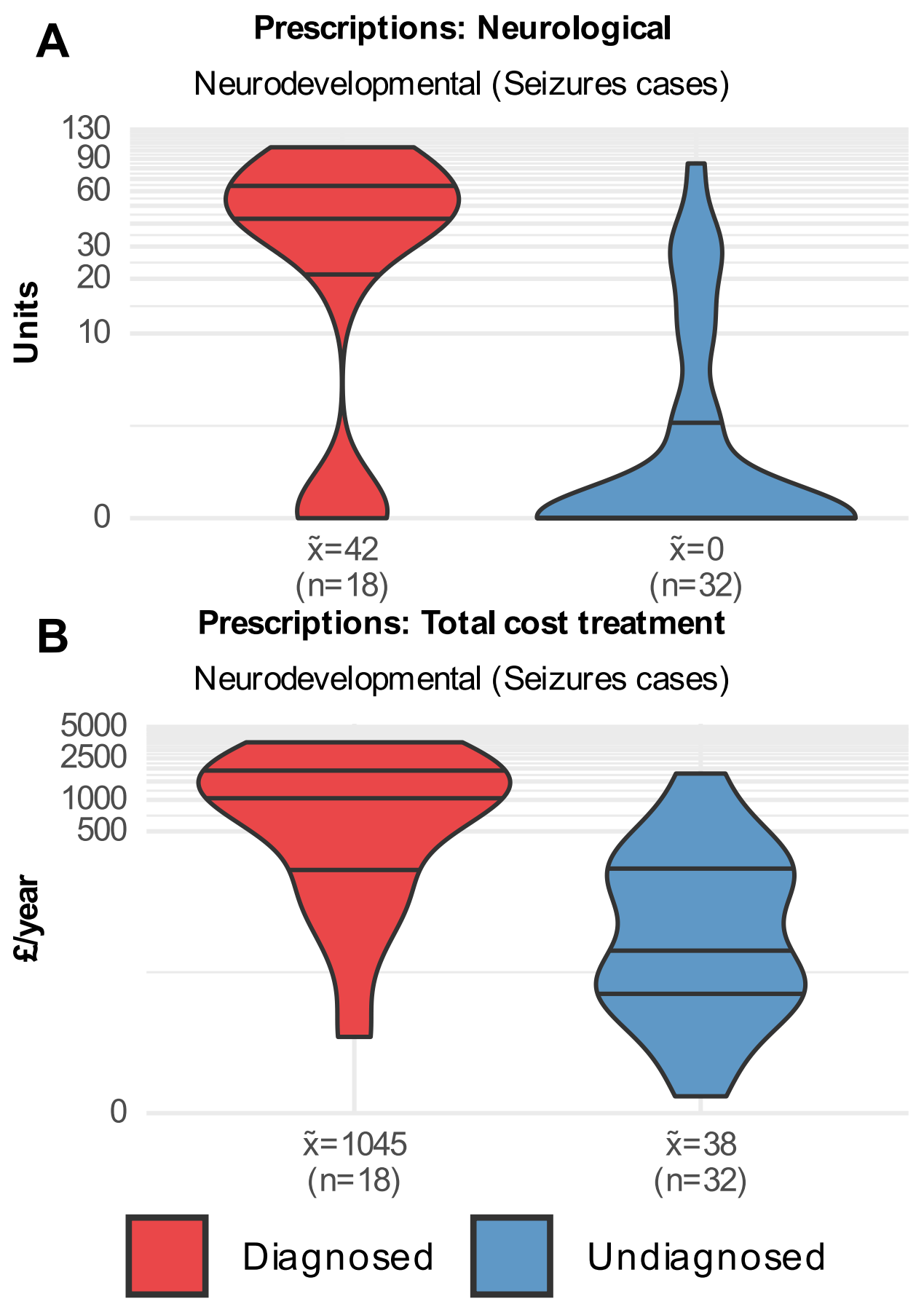

A-B: Prescriptions, highlighting statistically significant differences (n ≥ 7; p < 0.001) between diagnosed and undiagnosed groups across the Neurodevelopmental cases with Seizures. Each panel corresponds to a specific condition-utilization comparison, with violin plots contrasting diagnosed (red) and undiagnosed (red) groups. The y-axis uses a pseudo-log scale to accommodate wide-ranging values. Median, interquartile range (IQR), and sample size for each group are annotated below the respective plots.

© 2026 Dias JML et al. *JAMA Network Open*.

eFigure 6. Violin Plot Visualization of Health Care Utilization: Critically Ill Prescriptions

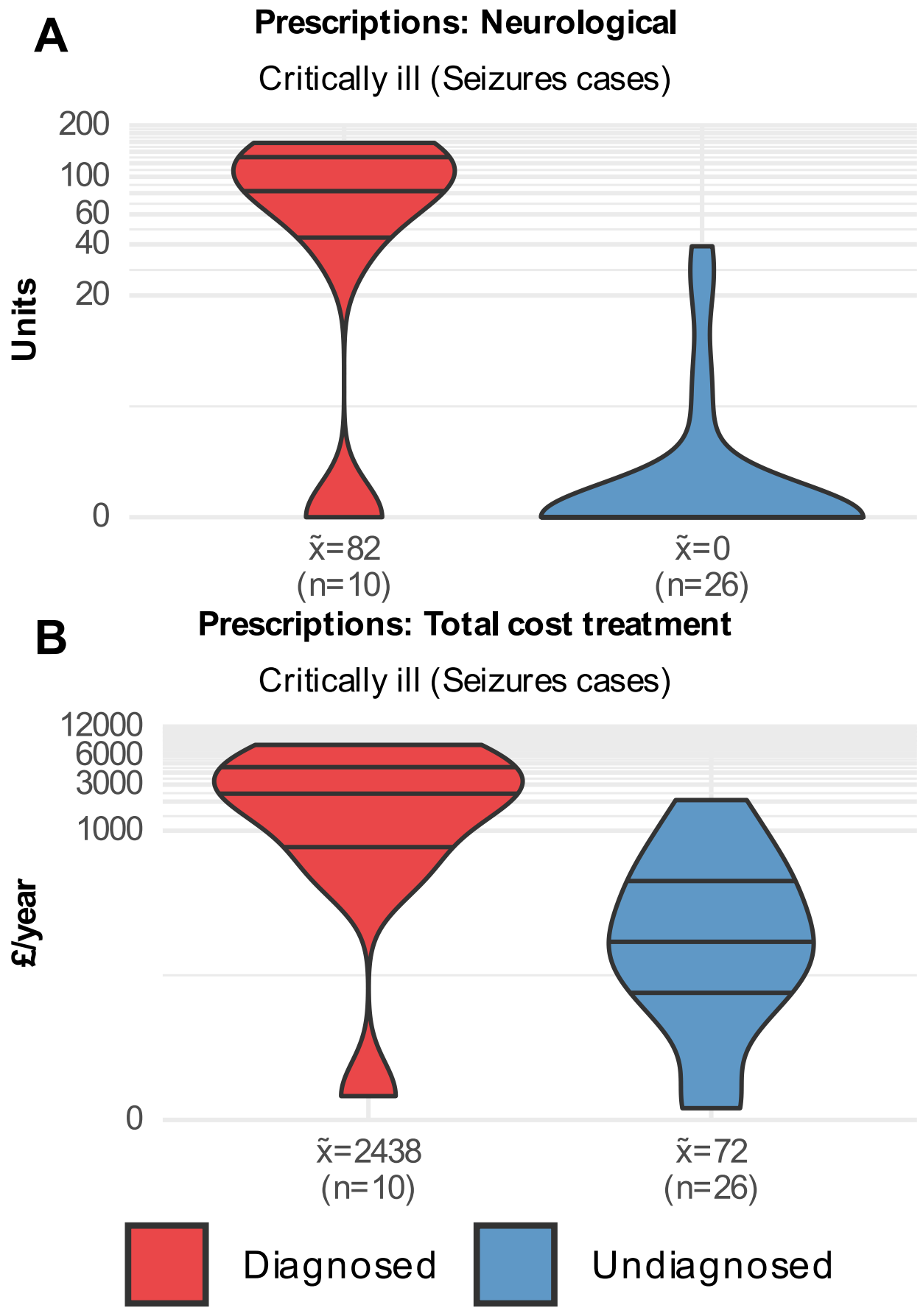

A-B: Prescriptions, highlighting statistically significant differences ($n \geq 7$; $p < 0.001$) between diagnosed and undiagnosed groups across the Critically Ill cases with Seizures. Each panel corresponds to a specific condition-utilization comparison, with violin plots contrasting diagnosed (red) and undiagnosed (red) groups. The y-axis uses a pseudo-log scale to accommodate wide-ranging values. Median, interquartile range (IQR), and sample size for each group are annotated below the respective plots.

eFigure 7. Predictive Modeling of Genetic Diagnosis

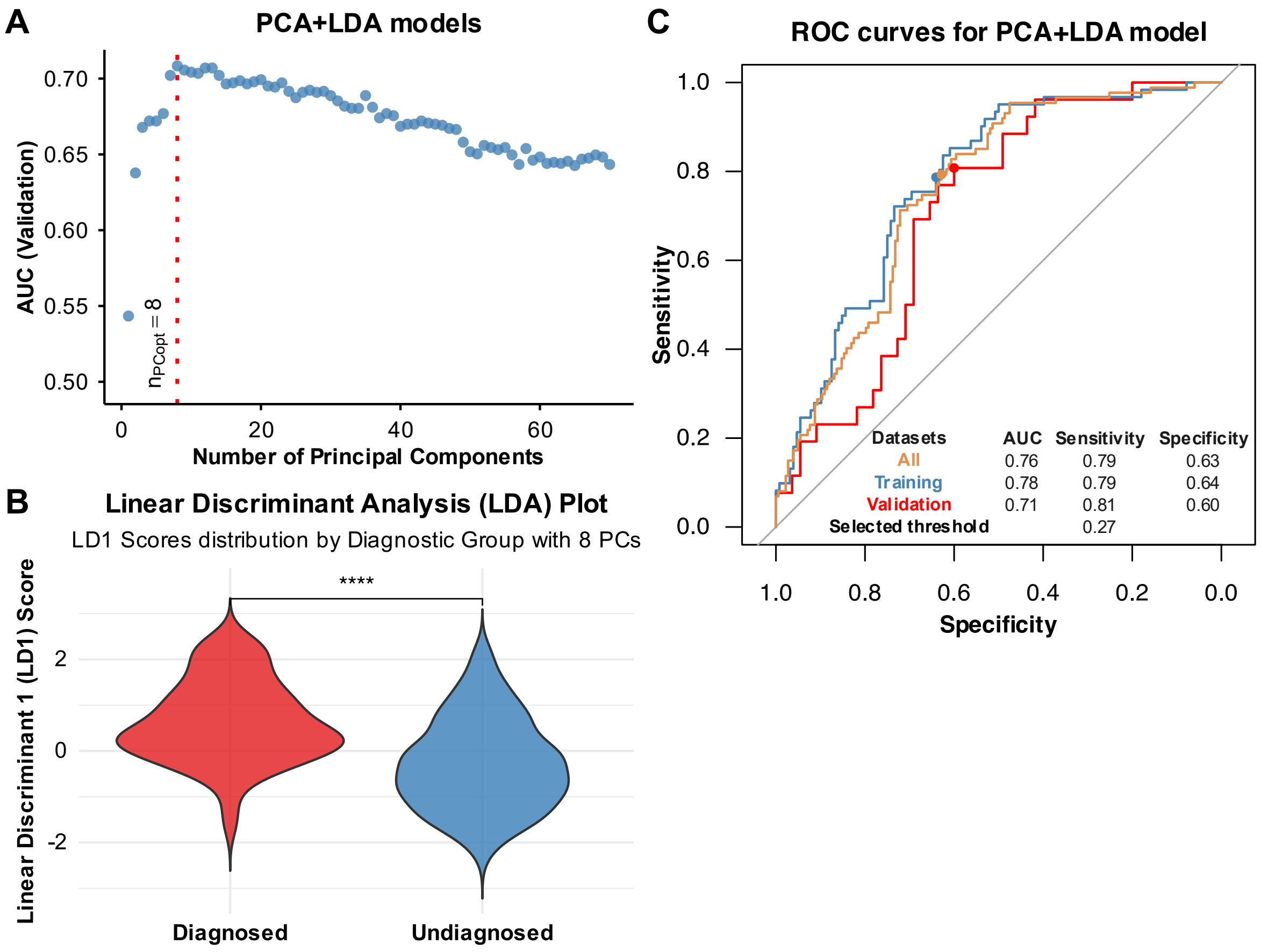

A: Average AUC on the validation set as a function of the number of principal components (PCs) for the PCA+LDA models. The red dashed line marks the number of PCs that achieved the highest model performance. B: Violin plots that reveal differences between the diagnostic categories using LDA evaluation of HPO terms, demographics and primary care records. Mann-Whitney U test: p<0.0001, indicating significant group differences. C: ROC curves for the PCA+LDA model, developed using the optimal number of PCs. The curves for the full (orange), training (blue), and validation (red) datasets are shown. Each curve represents the bootstrap model iteration with an AUC closest to the average AUC from all iterations. The point on each curve indicates the optimal classification threshold that maximizes the sum of sensitivity and specificity.

© 2026 Dias JML et al. *JAMA Network Open*.
