# Supplementary material for: Diagnosis Through Whole Genome Sequencing and Care Utilization in Children With Severe Illness
Source: JAMA Netw Open. 2026 Sep 17;9(9):e2634372. doi: 10.1001/jamanetworkopen.2026.34372 (PMC13586901; doi:10.1001/jamanetworkopen.2026.34372)
Supplement: Supplementary file 2 [file jamanetwopen-e2634372-s003.pdf]

Data Sharing Statement

Dias. Long-Term Secondary Care Utilization in a Survivor Cohort of Children and Young People With Serious Illness. *JAMA Netw Open*. Published September 17, 2026. doi:10.1001/jamanetworkopen.2026.34372

Data
Data available: No

Additional Information
Explanation for why data not available: As per terms of the ethics; data are reposited in the NIHR Rare Diseases Bioresource. Researchers can make an application for access.
